# Retinal Microstructural Changes in Aging Rhesus Macaques

**DOI:** 10.1002/alz70856_103443

**Published:** 2025-12-25

**Authors:** Yin Allison Liu

**Affiliations:** ^1^ UC Davis, Sacramento, CA, USA

## Abstract

**Background:**

Human studies have demonstrated progressive retinal thinning with aging, and rhesus macaques undergo similar physiological and behavioral changes. Alterations in their maculae can be visualized non‐invasively and with high resolution using optical coherence tomography (OCT). This study aims to evaluate the retinal microstructural changes in aging rhesus macaques as measured by OCT.

**Methods:**

We obtained spectral‐domain OCT images from 36 eyes of 18 geriatric rhesus macaques (12 females, mean age 22±3 years) using the Heidelberg Spectralis®. A customized segmentation algorithm was employed for automatic retinal layer thickness measurements, which were then manually corrected for accuracy. The layers measured included the retinal nerve fiber layer (RNFL), ganglion cell layer‐inner plexiform layer (GCL‐IPL), inner nuclear layer (INL), outer plexiform layer (OPL), outer nuclear layer (ONL), inner segment (IS), outer segment (OS), retinal pigment epithelium (RPE), and total retinal thickness (TRT). Simple linear regression and descriptive statistics were performed using Prism GraphPad to analyze the relationship between retinal layer thickness and age.

**Results:**

TRT had the strongest significant association with age and retinal thickness (β=‐1.84, 95% CI: ‐3.18 to ‐0.51). GCL‐IPL (β=‐0.62, 95% CI: ‐1.18 to ‐0.05), INL (β=‐0.55, 95% CI: ‐0.82 to ‐0.28), and RNFL (β=‐0.45, 95% CI: ‐0.81 to ‐0.09) also showed significant associations. OPL (β=‐0.02, 95% CI: ‐0.16 to 0.13), ONL (β=‐0.36, 95% CI: ‐0.93 to 0.21), IS (β=‐0.06, 95% CI: ‐0.27 to ‐0.14), OS (β=0.15, 95% CI: ‐0.14 to 0.44), and RPE (β=0.06, 95% CI: ‐0.24 to 0.36) were nonsignificant.

Statistically significant age‐related decreases were observed in the RNFL (*p* = 0.016), GCL‐IPL (*p* = 0.033), INL (*p* = 0.0002), and TRT (*p* = 0.008) suggesting that these layers progressively thin with age.

**Conclusion:**

Rhesus macaques experience age‐related retinal thinning like humans which may serve as markers of aging. Future studies should use a larger cohort of macaques and include behavioral correlations to identify potential retinal biomarkers for Alzheimer's Disease.

